# Accelerated Breeding for *Helianthus annuus* (Sunflower) through Doubled Haploidy: An Insight on Past and Future Prospects in the Era of Genome Editing

**DOI:** 10.3390/plants12030485

**Published:** 2023-01-20

**Authors:** Londiwe M. Mabuza, Nokuthula P. Mchunu, Bridget G. Crampton, Dirk Z. H. Swanevelder

**Affiliations:** 1 Biotechnology Platform, Agricultural Research Council, Onderstepoort Campus, Onderstepoort, Pretoria 0110, South Africa; 2 Department of Plant Sciences, Faculty of Natural and Agricultural Sciences, University of Pretoria, Private Bag X20, Pretoria 0028, South Africa; 3 Strategy, Planning and Partnerships, National Research Foundation, Pretoria 0184, South Africa

**Keywords:** sunflower, doubled haploid, genome editing, CRISPR/Cas9, *CenH3*

## Abstract

The aim of any breeding process is to fully express the targeted, superior/desirable parent characteristic in the progeny. Hybrids are often used in this dynamic, and complex process for which homozygous parents—which may require up to eight generations of back crossing and selection—are required. Doubled haploid (DH) technologies can facilitate the production of true breeding lines faster and in a more efficient manner than the traditional back crossing and selection strategies. Sunflower is the third most important oilseed crop in the world and has no available double haploid induction procedure/technique that can be efficiently used in breeding programs. A reproducible and efficient doubled haploid induction method would be a valuable tool in accelerating the breeding of new elite sunflower varieties. Although several attempts have been made, the establishment of a sunflower doubled haploid induction protocol has remained a challenge owing recalcitrance to in vitro culture regeneration. Approaches for haploid development in other crops are often cultivar specific, difficult to reproduce, and rely on available tissue culture protocols—which on their own are also cultivar and/or species specific. As an out-crossing crop, the lack of a double haploid system limits sunflower breeding and associated improvement processes, thereby delaying new hybrid and trait developments. Significant molecular advances targeting genes, such as the *centromeric histone 3* (*CenH3*) and *Matrilineal* (*MTL*) gene with CRISPR/Cas9, and the successful use of viral vectors for the delivery of CRISPR/Cas9 components into plant cells eliminating the in vitro culture bottleneck, have the potential to improve double haploid technology in sunflower. In this review, the different strategies, their challenges, and opportunities for achieving doubled haploids in sunflower are explored.

## 1. Introduction

The global oilseed market has emerged as one of the most competitive vegetable markets in the world owing to genetic improvement and advancement of cropping systems [1]. During the period between 2014–2018, *Helianthus annuus* L. (sunflower) accounted for 9% of the global oilseed market [1], maintaining its position as the third most important vegetable oilseed in the world following soybean and rapeseed [2]. Sunflower oil, however, due to its superior and preferred oil quality supersedes both rapeseed and soybean. Sunflower has great commercial significance on a global scale with a multitude of uses ranging from seed oil and flour for human consumption, oilseed cake for animal feed purposes, raw material for oleo chemistry, to a biofuel feedstock source [3]. Sunflower production is hindered by numerous biotic challenges, including broomrape, sunflower stem weevil, European sunflower moth, *Alternaria* leaf spot, downy mildew, *verticillium* wilt, phomopsis, *Sclerotina*, and rust [4,5,6]. Furthermore, climate change threatens to be accompanied by various limitations to crop production, including unpredictable temperature fluctuations, drought, emergence of novel diseases, and pests [7]. These pressures require accelerated breeding pipelines to ensure increased genetic gain in crucial crops. Conventional, mutation breeding and accelerated breeding technologies, such as haploidization (doubled haploids), have been able to address some of these challenges through the production of resistant cultivars [8].

Breeding is an essential part of crop improvement and breeding programs for the development of sunflower hybrids have been on-going for over half a century [9]. The pioneering sunflower breeding work started in Russia during the 1960s to develop varieties with increased oil content [9]. This was followed by the development of cytoplasmic male sterility (CMS) involving a cross between *Helianthus petiolaris* and cultivated sunflower (Leclercq 1969 as referenced by [9]). This work resulted in the release of the first sunflower hybrid during the 1970s [10]. The primary aim of any breeding program is the homogenous expression of the desired phenotype in the progeny [11]. Sunflower is an insect, open-pollinated species, and therefore, undesirable genetic and phenotypic-variation associated heterogeneity due to random crosses may arise [12]. Furthermore, conventional breeding requires considerable space and resources for plant selection [7]. This can cause breeding programs to take up to a decade to produce a new line of sunflower [3]. Constraints associated with heterogeneity in lines can be eliminated by establishing true breeding lines. True breeding lines can be obtained by repetitive backcrossing to the parental line containing the desirable trait and progeny selection or by developing haploids, with subsequent chromosome doubling to form doubled haploid (DH) lines [13]. Although sunflower doubled haploids have been previously applied in breeding programs [4], the absence in sufficiently diverse genetic resources and its recalcitrance to in vitro transformation have been a hindrance in the development of a doubled haploid induction procedure that could be efficiently used in breeding programs [14,15]. As an out-crossing crop, the lack of a double haploid system limits sunflower breeding and associated improvement processes, thereby delaying new trait developments. 

Sunflower has a haploid (2 n = 2 x = 34) genome of approximately 3.8 Gb [16,17]. The sunflower whole genome and pan genome sequencing projects have recently been completed [16,17], and this provides new opportunities for sunflower yield and quality improvement. Furthermore, recent advances in in vivo haploid induction technology through mitotic/meiotic process manipulations and genome editing promise to provide solutions for fast tracked sunflower breeding. In this review, past and present haploid induction methods in respect to sunflower are looked at, highlighting hindrances and opportunities for attaining an efficient sunflower haploid induction method in this new era of targeted genome editing. 

## 2. Doubled Haploids: Induction Methods and Their Role in Plant Breeding and Crop Improvement

**Figure 1:**
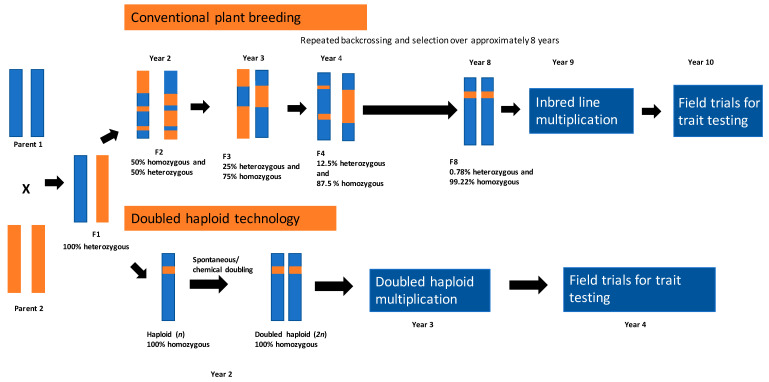
Comparison of conventional breeding and doubled haploid (‘accelerated breeding’) technology breeding methods (Illustration adapted from [21] 2022).

Haploids are plants carrying a genome derived from a single (haploid) gamete (i.e., sperm or egg cell), i.e., a cell with a single chromosome set [13,18]. Haploid plants are unable to go through meiosis and are therefore sterile [19]. Fertility is restored through chemically induced, or spontaneous, chromosome doubling, resulting in 100% homozygosity in a single generation [11,19]. The resulting doubled haploid (DH) line removes the need for backcrossing to a desirable parent line over numerous generations, thereby expediting true-breeding line production significantly (Figure 1) [18,20].

Additionally, DHs can be used to accelerate pyramiding multiple mutants, forward mutagenesis screening, downsizing ploidy levels (e.g., tetra- to diploid), the generation of homozygotes for gametophyte-lethal mutations, and reducing inbreeding depression associated with self-pollination [19,20]. Doubled haploids can also be employed to rapidly generate mapping populations, i.e., chromosome substitution lines [18]. Naturally occurring haploids have been reported for several cereal crops, including rice, wheat, and maize, [13] but to our knowledge, not for sunflower. The recovery frequency of these is, however, too low for practical applications and therefore difficult to manage in breeding programs [11].

Efforts to produce doubled haploids have been made for numerous oilseed species, including sunflower (Table 1), with only a few successes that have been adopted for crop improvement [22,23]. Technologies for haploid induction are available for less than thirty plant species, which include maize, oats, rice, and wheat [13,22]. These technologies include pollen irradiation, twin embryo seed selection, alien cytoplasm, sparse pollination, wide hybridization, and microspore culture [22,24]. Both microspore culture and hybridization were demonstrated as the most successful methods and have become the foundation of modern doubled haploid technology in plant breeding [18,23].

Microspore culture haploid induction technologies include androgenesis, i.e., the generation of haploids from male gametic material (paternal haploid induction) [19]. Androgenesis involves in vitro cultivation of immature anthers to allow the conversion of the developmental pathway of immature pollen grains from gameophytic to sporophytic [19]. Androgenesis is well established in haploid induction for plant breeding purposes and has been successfully demonstrated in species, such as *Brassica* species, solanaceae species, *Triticum aestivum* (wheat), and *Zea mays* (maize) [19]. Gynogenesis (maternal haploid induction) is haploid induction through the production of haploid embryos from unfertilized female gametophytes [19,24]. This is achieved through in vitro culture of un-pollinated whole flower buds or flower parts, such as ovules, ovaries, and the placenta. Although haploid induction through gynogenesis (Table 1) has been achieved in various species, including sunflower, its application in plant breeding is limited to *Beta valguris* L (Sugar beet) and *Allium cepa* (Onion) [19]. 

Wide hybridization is a widely demonstrated haploid induction method that involves interspecific or intraspecific crosses that result in the selective loss of the parental chromosomes [18,22]. Selective hybridization haploid production was first demonstrated in barley (Kasha and Kao, 1970 as referenced by [22]) where crosses between *Hordeum vulgare* and *Hordeum bulbosum* resulted in a haploid barley variety after embryo rescue since the hybrid endosperm caused abortion [24]. Other common examples of hybridization involve crosses between *Nicotiana tabacum* and *Nicotiana sylvestris*, which resulted in the loss of *N. sylvestris* chromosomes in the progeny and yielded haploid plants with only *N. tabacum* chromosomes [18,23]. This approach has been applied to cereal crops with great success, for example, a selective cross between wheat and maize [22,33], where maize chromosomes were eliminated resulting in haploid wheat progeny [22]. Wheat × pearl millet and pear × apple are also some examples of selective hybridization [23]. The eliminated chromosome’s *centromere histone 3* (*CenH3*) gene was either removed or smaller in size when compared to the retained chromosome, an association that provided insight on haploid induction via hybridization [22,24,34]. The development of haploid embryos through hybridization has been widely achieved in many species, including in a cross between sunflower and the genetically related species, lettuce, for haploid induction in lettuce [35]. The use of hybridization for haploid induction has not been explored extensively, and no data on sunflower haploid induction through hybridization exist in the literature [4]. 

The majority of these haploid technologies are time consuming, costly, genotype-dependent, and have too low efficiencies to be applied in sunflower breeding [23,36]. Doubled haploid induction methods, such as microspore culture and irradiation of pollen grains, have been tested on sunflower with very little success [37] and are discussed in detail in the review by Blinkov et al. [4]. Although anther culture [27] and parthenogenesis [26] have been partially successful in the induction of haploids in sunflower, they are genotype specific and of limited use [4,15,37]. These limitations of anther culture and parthenogenesis techniques make them inappropriate for large scale haploid induction, especially since there are numerous genotypes involved in breeding programs. 

### Haploid Induction by Meiotic/Mitotic Process Manipulations e.g., Centromeric Histone Protein 3 (CenH3) Modification

**Figure 2:**
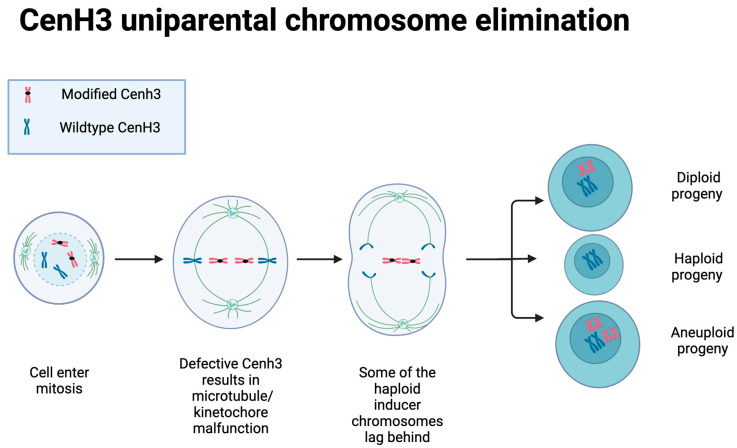
Illustration of centromere function during zygote mitosis. A plant with modified *CenH3* gene is crossed with a wild-type plant resulting in uniparental chromosome elimination leading to haploid progeny and/or incomplete elimination of haploid inducer genetic material leading to aneuploid progeny (illustration adapted from Chan, 2010). Created with BioRender.com (accessed on 11 January 2023).

Modification of phospholipase coding in genomic regions, *phospholipase-a1* (*PLA1*)/*Matrilineal* (*MTL*), *Not like dad* (*NLD*), and the *domain of unknown function 679 membrane protein* (*ZmDMP*) in major cereal crops, such as rice and maize, has been identified as responsible for haploid induction [38]. These genes are responsible for facilitating normal fertilization of egg cells, and mutations in these genes therefore trigger haploids upon egg fertilization failure resulting in parthenogenesis [39]. These gene homologs have, however, only been identified in monocotyledonous plants, except for the *ZmDMP*, which has also been identified in several dicotyledonous species [40]; to our knowledge, these gene homologs are yet to be identified in sunflower. Ravi et al. [41] demonstrated a centromere modification method in *A. thaliana* through which haploid inducer plants were produced. In this method, a *centromere histone 3* null mutant (*Cenh3*), with a mutated version of the CenH3 protein, was crossed with a wild-type plant that resulted in only the wild-type plant’s chromosomes, with functional CenH3 proteins, being transferred to the progeny and none of the mutant’s (Figure 2) [42]. Incomplete uniparental chromosome elimination can occur, however, leading to a percentage of aneuploid progeny (Figure 2). Due to the universal structure and function of the CenH3 protein in plants, the use of a gene editing haploid induction method promises wide applicability and success when compared to previously described haploid production methods [11]. Furthermore, the sunflower *CenH3* gene has been identified and characterized by Nagaki et al. [43]

The centromere is a crucial locus responsible for efficient and stable transfer of genetic material from gametes to embryos by mediating sister chromatid segregation [44,45]. The centromere serves as a binding site for the kinetochore complex [11,46]. Spindle fibers bind to the kinetochore complex and pull chromatids toward opposite poles for proper segregation [46]. This process is facilitated by several centromeric proteins, including the centromeric histone protein (CenH3), which plays a critical role in chromosome segregation [11]. The CenH3 protein acts as an assembly point for other kinetochore proteins [43]. The CenH3 protein is exclusively localized to functional centromeres, which is a key feature in distinguishing the centromere from the surrounding pericentromere [18,44]. It has been suggested that the CenH3 protein is the only protein required for fully functional centromeres [46]. 

**Figure 3:**
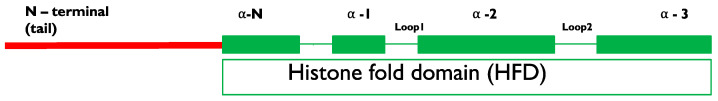
Typical schematic structure of the plant *centromeric histone protein 3* (*CenH3*) gene.

The CenH3 protein consists of two domains: A highly variable N-terminal and a highly conserved C-terminal histone fold domain (HFD) (Figure 3). The N-terminal is variable in both sequence and length and may even vary within species [11,47], while the HFD is conserved across species [48]. The N-terminal contains a single alpha helix (Figure 3) while the HFD region contains three alpha helices (α-1, α-2, α-3) separated by two loops (Figure 3). The HFD part of the CenH3 has been identified as the essential part of centromere localization during mitosis, even in the absence of the N-terminal. However, the variable N-terminal tail was observed to play a critical role in meiosis [41,45,49]. The N-terminal tail is also responsible for recruitment and stabilization of centromere complex proteins [50]. 

There are currently two approaches for applying the CenH3–haploid inducer workflow. The first method is based on a two-step approach where a ‘mutant tailswap’ is used to rescue modified *CenH3* transgenes as described by [41]. In this approach, genetically modified *CenH3* variants must be cloned and partially expressed to complement the knockout mutation [11,33]. The complementation of the hypervariable N-terminal with histone H3.3 fused with the green fluorescent protein (GFP) in *Arabidopsis thaliana* resulted in sterile plants with meiotic defects and crossing with wild-type plants resulted in chromosome mis-segregation and elimination of the mutant’s genetic material [41]. The ‘tailswap’ method resulted in haploid progeny at a frequency between 25–45% when the inducer line was maternal in *Arabidopsis*. This has been tested in some crop species but resulted in relatively low efficiencies, i.e., 0.065–0.86% haploid progeny in maize, 0.2–2.3% in tomato, and 0.3–1% in rice [24]. In hexaploid wheat containing three copies of the *CenH3* gene, namely *CenH3α-A*, *B*, and *D*, mutations with amino acid changes in the N-terminal tail called the restored frameshift (RFS) in *CenH3α-A* were induced together with a knockout of *CenH3α-B* and *CenH3α-D*. This triggered paternal haploid induction with frequencies of up to 8% [50]. This approach has been used in cereal crops, such as maize, wheat, and in *Brassica* [40,50,51]. 

The second approach is a one-step method based on the targeting of the endogenous *CenH3* gene by *CenH3* gene silencing using Ribonucleic acid interference (RNAi), randomly induced point mutations, and gene knockouts CRISPR/Cas9 [11]. Kuppu et al. [47] and Karimi-Ashtiyani et al. [20] presented a haploid induction method that studied a variety of ethyl methane sulfonate (EMS)-induced mutations in the conserved histone fold domain (HFD) regions of the *CenH3* gene in *Arabidopsis*, barley, and sugar-beet, which resulted in haploid inducers (when out-crossed with wild-type *CenH3* plants). Random point mutations on five amino acids—P82S, G83E, A132T, A136T, and A86V—produced paternal haploids at a rate of 0.61% to 12.2% that were normal in appearance and fully fertile when self-fertilized [20,47]. This indicates that the *CenH3* from the mutant plants remained functional but incapable to compete with the normal ‘wild-type’ *CenH3*s [23,47]. Further approaches have demonstrated complete deletions of the α-N helix of the HFD region resulting in haploids upon crossing with wild type in *A. thaliana* [52]. This single-step method provides ease to the previously described ‘tailswap’ method, which combined a chimeric transgene with a gene knockout that may be difficult to engineer, and the applicability may be reduced [23,47]. The idea behind the *CenH3* modification method is that interference with the centromere structure may result in a less competitive or weak centromere when confronted with a non-modified or wild-type centromere [11]. The *CenH3* haploid inducer methods result in non-transgenic haploid plants as the chromosomes of the transgenic haploid inducer plant are eliminated [33]. 

## 3. Methods for *CenH3* Modification

Various gene editing tools have been utilized to achieve modification of the *CenH3* gene for haploid inducer line production, including RNAi, random chemical mutagenesis (Ethyl methane sulfonate, EMS), and more recently, CRISPR/Cas9 [20,40,41,47,50]. Ribonucleic acid interference (RNAi) is a naturally occurring mechanism used by eukaryotic organisms for regulation of transposable elements, destruction of invading foreign viruses, and prevention of homologous chromosomes [53]. RNAi for artificial gene silencing is based on the production of homologous double-stranded RNA (dsRNA) homologous to the target gene with hairpin formation [54]. This dsRNA is processed into small interfering RNAs (SiRNAs, 21–24 nucleotides), which provide target sequence specificity of an endonuclease that facilitates the degradation of homologous RNA sequences [54]. RNAi was one of the first methods utilized for *CenH3* modification for the purpose of haploid induction in plants and has been used for *CenH3* modification in *A. thaliana* [41,49] and banana [53]. RNAi has also been previously used for gene silencing in sunflower to induce resistance against the *Tobacco streak virus* [55]. RNAi is however inefficient, often results in incomplete gene silencing [53], and does not result in permanent mutations [56].

Alkylating agents contribute 80% of the total number of mutagens used in plants [57]. Alkylation involves the substitution of an alkyl group (C_2_H_5_) with hydrogen in the nitrogen bases. Alkylating agents normally give rise to ‘base-pair’ substitutions where alternate bases are incorporated during replication [58]. These agents’ bond with guanine (G) in the DNA sequence to form an abnormal alkyl–guanine base that is recognized as an adenine (A) instead of a G, which results in a transition mutation [57]. Ethyl methane sulfonate (EMS) is the most widespread alkylating agent since it is easy to use and handle and is effective [58]. Chemical mutagens are generally considered to be more efficient and specific than physical mutagens [59], but both could be used simultaneously to enhance mutation frequency [60]. An existing population of EMS mutagens were screened for amino acid changes in the *CenH3* histone fold domain region and successfully used as haploid inducer lines in *Arabidopsis* [20,47,52]. A sunflower EMS population was recently studied for the presence of mutation in the *CenH3* that could be applied for haploid induction using the targeted induced lesions in genomes (TILLING) analysis method [61]; the identified mutations have however not been tested for haploid induction.

The clustered regularly interspaced short palindromic repeats (CRISPR) system is a defense mechanism that protects bacteria and archaea against viruses and other mobile genetic elements of foreign origin [62,63,64]. The CRISPR-associated protein (Cas)-mediated adaptive defense system in prokaryotes uses stretches of RNA sequences in combination with nuclease enzymes that cleave DNA at specific sites in invasive viruses, thereby suppressing or eliminating invasions [63,65]. This process is divided into three stages: Adaptation, expression, and interference. During adaptation, an insertion of the invading agent’s sequence into spacers known as CRISPR RNA (crRNA) of the bacterial CRISPR locus occurs, thereby providing the guide for targeting the virus with this immunity system [66]. A second RNA molecule, trans-activating RNA (tracrRNA), is responsible for recruiting the Cas9 endonuclease and binding onto the target DNA [65,67]. In the second stage, these two RNA molecules form a complex with the Cas nuclease, and the complex binds to the identified virus target site [66]. In the third stage, interference, the combined action of crRNA, tracrRNA and Cas protein results in the recognition and degradation of the viral target DNA [68]. 

**Figure 4:**
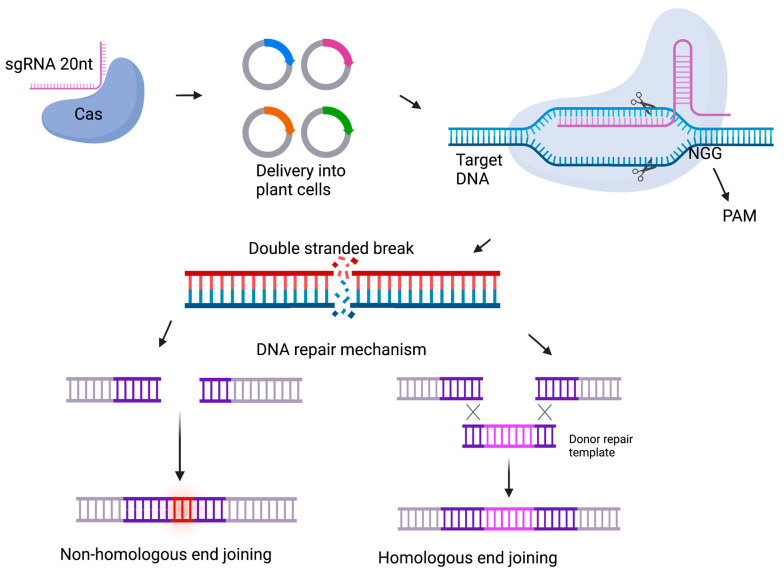
Schematic representation of the CRISPR/Cas9 genome editing mechanism in plant cells. Created with BioRender.com (accessed on 11 January 2023).

The bacterial CRISPR/Cas-based defense mechanism has been altered and developed to allow targeted double-strand DNA breaks in various organisms [63]. In the altered version of the CRISPR/Cas technology applicable for genome editing, the crRNA and tracrRNA have been combined and a single 20-nucleotide DNA molecule is added to form a small guide RNA (sgRNA or gRNA) [65]. The sgRNA and introduced Cas nuclease form a sgRNA/Cas complex (Figure 4), with the sgRNA necessary for target site recognition [69]. The sgRNA is associated with a short nucleotide sequence (approximately 3–6 nucleotides) known as the protospacer adjacent motif (PAM) (NGG in the case of *Streptococcus pyogenes*) with the PAM sequence depending on the bacterial species where the Cas9 enzyme was acquired from [62]. The presence of the PAM sequence downstream of the sgRNA activates the two Cas9 domains and leads to cleaving of the target site [70]. The Cas enzyme typically induces double stranded breaks approximately 4 nucleotides upstream of the PAM sequence [65]. CRISPR/Cas9 has been around for almost a decade, with the first successful experiment in plants reported in the generation of mutagenesis events in *Arabidopsis thaliana*, *Nicotiana benthamiana* and *Triticum aestivum* toward the end of 2013 [71,72]. 

CRISPR/Cas9 technology has been used for the majority of *CenH3* gene modification experiments [38,50,51,52,73,74,75]. This technology is more advantageous than other genome editing technologies as it does not require complicated designs and the assembly of DNA binding proteins, but it only requires a single Cas9 nuclease that can be programmed by engineering the guide RNA (gRNA) to direct target-specific cleavage [76]. Additionally, CRISPR can target methylated DNA, further increasing its applicability [77]. Target specific modifications driven by customizable direct nucleases, such as the clustered regularly interspaced short palindromic repeats (CRISPR) provide an effective tool to overcome the limitations that arise from random genome modification [78]. The main advantage of using induced mutagenesis technologies, however, is that they are often not considered to be genetically modifying (GM), and as such, they do not fall under GM regulatory and risk assessment frameworks of many countries [77]. Haploids produced through *CenH3* modification are, technically, non-GMOs—irrespective of the parent ‘haploid inducer’ line carrying a genetically modified *CenH3* gene as ‘haploid inducer’, since its chromosomes are lost during uniparental chromosome elimination [11]. 

Central to the success of these technologies viz RNAi and CRISPR/Cas9 is the need for genetic material to be stably delivered into target plant cells. Genetic editing of plants can be defined in two stages: The transfer of the genetic material into the cell and the integration of the genetic material into the genome [79]. The genetic material is either transiently expressed in plant cells, which is only for a short time, or permanently expressed [62]. The majority of genome engineering components are cloned into DNA plasmids prior to the introduction into plants; others however include RNA, proteins (e.g., designed nucleases), and ribonucleoprotein complexes [78]. Delivery of genome editing components can be challenging in plants [80]. It is therefore crucial to develop a specific and efficient delivery mechanism for a specific plant species or a line of interest. Given the pace at which routine genome editing is being adopted, more efficient genome editing methods are needed especially in crops, such as sunflower, that are difficult to transform [76]. Most of the genetic material delivery systems in place focus on the stable transformation of plants resulting in transgenic crops as opposed to transient expression. Delivery of genome editing reagents to intact plant cells is currently limited to particle bombardment, *Agrobacterium*-mediated transformation, and to some extent, viral delivery vectors [81]. According to our knowledge, there are currently no reports of CRISPR/Cas9 genome editing and only a single report on RNAi-based gene silencing in sunflower, and the difficulty in genome editing component delivery into sunflower cells could be a culprit. 

## 4. Delivery Methods for Genome Editing Components

Despite the possibilities provided by the CRISPR/Cas9 technology, it is yet to be routinely used in crop species [82]. This can be largely attributed to inherent challenges that come with the delivery and expression of recombinant proteins in plants, as well as a large system, which includes sgRNA and Cas9 enzyme gene [83,84]. The dependence on *Agrobacterium tumefaciens* for genetic transformation and delivery of components and tissue culture-based plant workflows and regeneration, including recalcitrance in several crops, including sunflower, add a host of limitations [85]. 

### 4.1. Agrobacterium Tumefaciens-Mediated Transformation

The genus *Agrobacterium* consists of plant pathogenic bacteria inhabiting the soil that cause crown gall disease characterized by tumors in infected plants [86]. This is achieved through a distinct plasmid that enables them to transfer a particular segment of their DNA (T-DNA) into the plant cells, resulting in the integration of this T-DNA into the plant host genome [78,87]. Initially, *Agrobacterium tumefaciens*-based transformation was achieved by infecting tobacco cells or protoplasts with *Agrobacterium tumefaciens* and regenerating genetically modified shoots from these cells [87]. *A*. *tumefaciens* contains a megaplasmid of almost 200 kb responsible for tumor induction and opine formation in infected plants [88,89]. This tumor-inducing (Ti) plasmid contains the T-DNA, delimited by repeats known as the left (LB) and right (RB) borders responsible for defining T-DNA boundaries [88]. The transfer of this plasmid is mediated by genes in the plasmid virulence regions namely *vir* genes [86,89]. Transformation plasmids were developed by disarming the tumor-inducing (Ti) plasmid through the removal of all the T-DNA genes to only allow the delivery of foreign DNA and allow the direct regeneration of transformed plants [78,87]. A majority of disarmed *A. tumefaciens* T-DNA plasmids typically contain *vir* genes, antibiotic resistance genes, LB and RB borders, and replication origin (*ori*) [88].

Components of interest can also be introduced into plants via a *A. tumefaciens*-mediated transient expression assay, often referred to as agroinfiltration, which has been mainly used to transiently express introduced components in dicotyledons [78]. Agroinfiltration was used for directly expressing proteins [87] and has an application in transiently expressing CRISPR-Cas components in plant cells to modify DNA regions without component integration into host DNA. *A. tumefaciens*-mediated delivery is one of the most widely adopted plant transformation methods. It is relatively affordable and easy to set up in the laboratory due to wide application and demonstration [78].

*Agrobacterium tumefaciens*-mediated transformation remains the method of choice in plant gene editing experiments, but transformation of sunflower remains a challenge with efficiencies of 1–4% [14,90,91]. Sunflower is associated with a host of transformation limitations, including low *A. tumefaciens* virulence, low transformation efficiencies, lack of stable integration of introduced genes, genotype dependence, and the production of chimeric plants among others [14,91]. Several studies have focused on the improvement of sunflower transformation, including the establishment of genotype-wide *A. tumefaciens* transformation protocols through testing various factors affecting plant transformation, such as *A. tumefaciens* titre, co-culture period, and plant vitamin treatments [91].

### 4.2. Particle Bombardment

Particle bombardment is another widely used technique for the delivery of gene editing reagents in plants [79,92]. Particle bombardment, also referred to as “delivery with a gene gun”, is a physical means for the facilitation of nucleic acid delivery in plants. This method is based on the bombardment of intact plant cells with DNA-coated high-velocity microprojectiles “shot” in with enough force to penetrate the target plant cells. The DNA will thereafter integrate into the host genome [78,79]. This delivery method targets intact plant material and therefore does not require protoplasts or cloning vectors, although they are often used [79,93]. Particle bombardment also allows for the delivery of multiple DNA constructs simultaneously [78]. A downside of this delivery system is that it often results in multiple copies of the transgene being integrated into the genome [60]. This may lead to numerous unwarranted effects, such as gene suppression and changes in gene expression levels in transgenic plants [78]. 

Particle bombardment has been used extensively in the generation of both commercial and experimental genetically modified crops [79]. Several research groups have used particle bombardment for sunflower transient transformation [94]. Particle bombardment has, however, also been used for the production of stable sunflower through a combination of bombardment and co-cultivation with *A. tumefaciens* [95,96]. These approaches demonstrated low efficiencies in transformation. 

### 4.3. Viral Vectors for Plant Genome Editing

Viral-based vectors have also been developed and employed in the delivery of genetic material in plants—although, this approach is more often used for mammalian cell transformation [97,98,99]. Over the past few decades, modified plant viruses have served as great vehicles for the expression of proteins and RNAs in plants for various functions [85,100]. On the introduction to a plant, the viral vector system will replicate and express the transgene of interest in the host [101]. The efficient machinery and comprehensive genome structure of viruses makes their genomes an excellent choice to be used as vectors [76,102]. The *tobacco mosaic virus* (TMV) was the first viral-based vector to be employed for virus-induced gene silencing (VIGS) in the model plant *Nicotiana benthamiana* [78], and numerous viral vectors have been explored from then on. 

Autonomously replicating virus-based vectors provide alternative means to deliver genome engineering (GE) components into plant cells [76]. Single-stranded (ss) DNA viruses, such as geminiviruses, have also widely been adopted as vectors for diverse crops [83]. These viruses can be modified to carry heterologous coding proteins [76]. However, due to cargo capacity constraints, these geminiviral replicons are usually utilized as “deconstructed” ssDNA viruses and only contain viral elements necessary for replication [103]. As a result, a majority of the studies on geminiviral replicons could either only deliver the sgRNA molecule and/or required *A. tumefaciens* transformation to facilitate systemic infection of the target cells. Recently, two independent studies on two geminiviral replicons, namely the *Bean yellow dwarf virus* (BeYDV) and *Beet curly top virus* (BCTV), were employed for the expression of the spCas9, sgRNA, LbCas12a, and its corresponding crRNA for successful genome editing and homology directed repair in *Nicotiana benthamiana* while retaining systemic movement after agroinfiltration [103,104].

Geminiviral vector replicons (GVRs), in particular, have the ability to increase gene targeting frequencies compared to traditional delivery systems, e.g., *Agrobacterium tumefaciens*, due to high viral load introduced into cells [80]. These viruses have a genome size of ±2.8 kb, usually with six overlapping open reading frames (ORFs) [76]. Geminiviruses are DNA based and have a wide host range, making them the ideal vectors [83]. They normally consist of two circular single-stranded genomes [105]; only one of these genomes is, however, required to initiate replication within a host [76]. The second is required for cell-to-cell movement within the plant host and is dependent on the presence of the former for its replication [105]. The use of geminiviruses for gene targeting has long been recognized, however, due to size limitations when constructing expression vectors; the viral gene responsible for movement is most often removed to accommodate the insert, thereby hampering efficient replication and restricting expression to the affected cells only [97]. Recently, two independent studies on two geminiviral replicons, namely the *Bean yellow dwarf virus* (BeYDV) and *Beet curly top virus* (BCTV), were employed for the expression of the spCas9, sgRNA and the LbCas12a, and its corresponding crRNA respectively for successful genome editing and homology-directed repair in *Nicotiana benthamiana* while retaining systemic movement after agroinfiltration [103,104]. These recent successes in virus-mediated tissue culture–free genome editing warrant further investigation, especially in transformation recalcitrant crops, such as sunflower.

## 5. Prospects for Doubled Haploids in Sunflower

Doubled haploids are a crucial aspect of enhancing and accelerating sunflower breeding [4]. The production of sunflower doubled haploids through gamma-induced parthenogenesis has facilitated the development of lines with resistance to downy mildew (*Plasmopora helianthi* Novot), phoma (*Phoma macdonaldii*), phomopsis (*Phomopsis helianthi*), Alternaria leaf spot (*Alternaria* spp) and broomrape (*Orobanche cumana*) [5,26,106], and herbicide tolerance (imidazolinone) [25]. Highly fertile F1 sunflower hybrids were also produced through anther culture [29] and used for heterosis breeding [4]. The production of haploids in sunflower is, however, limited to a small number of genotypes owing to bottlenecks, including recalcitrance to in vitro culture. Sunflower in vitro transformation studies and optimization protocols have been on-going since the early 1980s with minimal improvement [14]. Various factors play a critical role in the genetic transformation and in vitro generation efficiency in plants, and it may be difficult and tedious to single out individual factors as culprits for decreased transformation efficiency [14]. The low regeneration efficiency of sunflower has also impacted sunflower improvement and is revealed by the lack of a commercially available transgenic sunflower [14] and the delay in adoption of genome editing techniques, such as the CRISPR/Cas9 technology. Given the limitations presented by *A. tumefaciens* transformation in various plant species, analyzing the genomic factors affecting *A. tumefaciens* transformation need to be explored. Recently, a comparative transcriptomic study of *Agrobacterium tumefaciens* infection between tea (*Camellia sinensis* L.) and tobacco revealed the molecular basis for tea’s recalcitrance to genetic transformation [107]. Compounds present in tea, known as gamma-aminobutyrate (GABA) and catechins, affected plant–pathogen attachment, mineral acquisition, and quorum quenching, which could potentially impact *Agrobacterium tumefaciens*-mediated transformation of tea [107]. A similar study could offer beneficial information that could assist in the optimization of *A. tumefaciens*-mediated transformation in sunflower.

The *CenH3*-based haploid induction system has seen more failures than successes in crop plants, with failures in tomato, soybean [50], and carrot [73]. Further research on the CenH3 structure and function could assist in the enhancement of haploid induction in non-model crop plants. Furthermore, the reliance of *CenH3* modification techniques on tissue culture and plant transformation present a challenge on the adoption of this technique for recalcitrant crops, such as sunflower. Plant transformation is generally mediated by *A. tumefaciens* transformation or particle bombardment. Unfortunately, both these methods have reduced efficiencies because they only transform a small portion of the treated tissues, often require a viable plant regeneration tissue culture protocol and are quite tedious to work with [76].

The CRISPR/Cas genome editing platform has made, what was once the dream of many biologists, a reality: Pathogen-resistant, nutrient-rich crops, and doubled haploid crops can now be produced through single base pair and target-specific alterations to DNA. Even with these significant milestones, the CRISPR/Cas technology is not one without bottlenecks, i.e., in vitro regeneration dependencies. Tissue culture remains a labor intensive and time-consuming process even for crops with established regeneration protocols [108]. It can take up to a year to obtain a fully developed transformed plant, the development of a tissue culture–free protocol is therefore ideally required for effectively driving plant genome editing. Given the recent success using a geminivirus for systemic delivery of Cas9 and sgRNA as a single vector that successfully edited *N. benthamiana*, it is worth investigating other viral vectors for potential CRISPR/Cas delivery for genome editing especially in sunflower. Plant viral vectors hold great potential for accelerated genome editing and crop improvement due to their modest mechanism of delivery. Plants can be directly inoculated with viral vectors harboring genome editing components, and it can only take between 3–7 days for successful viral genome editing to take place allowing for rapid screening of a large quantity of plants [109]. The modification of the CenH3 could potentially affect plant fitness and reduce recovery of potential haploid inducer lines from in vitro regeneration. Virus vectors can deliver genome editing reagents to mature plants, and editing can occur before the onset of plant growth-limiting effects [109].

Considering the recent discoveries of molecular factors responsible for haploid induction e.g., *CenH3* gene mutations and uniparental chromosome elimination, and the advancement of gene editing tools, such as CRISPR/Cas9 and the use of viral vectors to eliminate the tissue culture bottleneck, an opportunity for haploid induction in sunflower has emerged. The success of future prospects in sunflower doubled haploid line production is dependent on the adoption and advancement of genetic manipulation technologies, such as the CRISPR/Cas9 system. The predicted decline in crop production influenced by evident climatic fluctuations makes it crucial for accelerated breeding techniques, such as doubled haploidy to be adopted to ensure sustainable food production.

## Figures and Tables

**Table 1:** Doubled haploid induction methods used in oilseed breeding.

Plant Species	Haploid Induction Method	Application	References
Sunflower (*H. annuus*)	Parthenogenesis	Resistance to broomrape, phoma, imidazolinone, and downy mildew	Drumeva [5,25]; Todorova [26]
	Anther culture	Fertility restoration	Bohorova [27]; Saji and Sujatha [28]; Jonard and Mezzarobba [29]
*Brassica napus* L. (Rapeseed)	Spontaneously occurring	Oil yield enhancement	Reviewed by Kucera et al. [30]
*Brassica* spp.	Microspore culture/embryogenesis	Modification of fatty acid profiles, fatty acid levels, and drought tolerance	Reviewed by Ferrie et al. [31];Daurova et al. [32]

## Data Availability

Not applicable.
